# Mind the gap: Standardising preclinical testing of bone-repair biomaterials

**DOI:** 10.1016/j.bbiosy.2025.100121

**Published:** 2025-09-09

**Authors:** Anders Palmquist, Furqan A. Shah

**Affiliations:** Department of Biomaterials, Sahlgrenska Academy, University of Gothenburg, Gothenburg, Sweden

**Keywords:** Bone-repair biomaterials, Bone structure, Bone quality, Preclinical evaluation

## Abstract

The use of bone-repair biomaterials is rapidly expanding to meet the needs of an ageing and increasingly active population, often with compromised bone quality. However, inconsistencies in how materials are assessed preclinically, across animal models, sampling strategies, and analytical techniques, have led to flawed comparisons and misleading claims. Fundamental differences in material properties and the biological responses they elicit are frequently ignored, conflating distinct mechanisms of bone formation. This "*apples vs. oranges*" problem is magnified by the growing diversity of biomaterials. Here, we call for a more systematic, context-aware approach to biomaterial evaluation that emphasises standardisation and biological relevance.

## Highlights

• Bone-repair biomaterials are classed as osteoconductive, osteoinductive, and bioinert.
• Materials differ in degradation behaviour, bioactivity, and bone formation mechanisms.
• Flawed interpretations arise from inconsistencies in preclinical testing strategies.
• Differences in the biological responses elicited by implant biomaterials are ignored.
• Biologically-informed, context-aware approach to biomaterial evaluation is essential.

## 1 Bone-repair biomaterials

Bone-repair biomaterials are frequently used in clinical disciplines to restore or replace anatomical structure and function due to trauma, disease, and congenital disorders. A variety of materials with a wide range of surface properties are used as temporary or permanent implants, with generally good clinical performance. However, preclinical research is increasingly focussed on the next generation of bone-repair biomaterials, leveraging advanced manufacturing techniques such as additive manufacturing and bioprinting, novel material compositions and surface modifications, incorporation of therapeutic agents, and approaches that mimic biological processes [1]. However, inconsistencies in animal models, experimental controls, and analysis strategies create interpretational pitfalls. As bone-repair biomaterials become increasingly diverse in composition and behaviour, the need for valid cross-material comparisons is more pressing than ever. Yet, the lack of standardised, biologically-informed region(s)-of-interest (ROIs) selection undermines many such comparisons, resulting in misleading conclusions and hindering the clinical translation of promising materials. Here, we aim to highlight the need for improved study design and analytical strategies to avoid "*apples vs. oranges*" comparisons.

## 2 Bone is hierarchical, heterogeneous, and dynamic – the case for spatially resolved analysis

The extracellular matrix (ECM) of bone is a nanocomposite of organic and inorganic constituents, including type-I collagen, non-collagenous proteins, and ion-substituted carbonated apatite [2,3]. Osteocytes–highly dendritic and intercommunicating cells–are embedded within the ECM, where both the osteocyte density and interconnectivity decline with age [4]. Recent advances in 3D imaging, such as synchrotron radiation nano-computed tomography, now enable quantitative characterisation of the osteocyte lacuno-canalicular network, revealing morphology–permeability relationships linked to osteocyte mechanosensation and remodelling, with permeability shown to decline with age [5,6]. The spatial arrangement of the osteocytes is intricately linked to the structural organisation of the surrounding extracellular matrix [7]. Moreover, variations in lacuno-canalicular network architecture modulate the local mechanosensitivity [8]. Osteocytes are also important in peri‑implant bone [9]. These extracellular and cellular components give rise to a multiscale, hierarchical architecture spanning the macro-micro-nano continuum [10]. Furthermore, bone is a dynamic tissue, undergoing targeted and untargeted remodelling for damage repair, renewal, and adaptation to load [11]. Until a given volume of bone is renewed, continued mineral maturation is measured as increase in crystallinity (*i.e.*, the *size* and *perfection* of the crystal lattice) and carbonate ion (CO_3_^2-^) content [[12], [13], [14]]. Collagen also displays a progressive increase in crosslinking [15]. From a *materials science* perspective, the chemical and mechanical properties (of any given location) of bone vary and evolve as a function of time, becoming more brittle with age. Thus, spatially-resolved (site-specific) rather than bulk analysis is required.

## 3 Sampling appropriate region(s)-of-interest in peri‑implant bone

Many specialised nano-analytical techniques, *e.g.*, electron tomography and atom probe tomography, allow probing of only very small volumes. Therefore, precise selection of the ROIs is critical and should be linked with complementary techniques capturing larger fields of view [16]. Yet, there are many challenges in standardising analytical procedures and selecting appropriate ROIs. The chemical and physical properties of bone-repair biomaterials, including degradation behaviour, directly contribute to this dilemma. There are two major aspects to consider: (*i*) whether the external form of a biomaterial remains unaltered over time *in vivo*, and (*ii*) if a biomaterial possesses the ability to spontaneously achieve bone formation at non-bone anatomical sites [17].

Bone quality parameters such as ECM composition, microstructure, and mechanical properties are typically evaluated to characterise the *in vivo* performance of implant materials. For unbiased comparisons of osteogenic potential, it is essential to analyse bone sites of similar chronological age and/or biological maturity. The healing environment in bone contains contributions from poorly organised ‘woven’ bone and highly ordered ‘lamellar’ bone, which differ in terms of mineral particle thickness, the degree of mineral particle alignment, and the mechanical properties of the ECM [[18], [19], [20]], attributable to differences in collagen fibril organisation. Even in highly remodelled areas, interstitial bone is generally older than the osteonal lamellae [21]. One way to assess tissue age is with calcium-binding fluorescent dyes that label active mineralisation fronts [22]. These labels can later be spatially correlated with specific bone regions, offering insights into the maturation timeline of peri‑implant bone.

In many studies, ROIs are defined at a fixed radial distance, *i.e.*, “ROI at X distance”, from the implant surface. While this may offer geometric consistency, it does not account for material-specific differences in bone formation dynamics. For instance, comparing "bone at 20 µm" from the implant surface of both titanium and a fast-resorbing magnesium implant may yield tissues of markedly different age or biological origin. Around titanium, which remains dimensionally stable over time, the peri‑implant bone is typically older and more mature, whereas the same distance from a degrading magnesium implant may correspond to newly formed bone infilling the regions vacated by the resorbed material [23]. Without recognising these context-specific differences, titanium may appear more conducive to bone maturation simply because the surrounding tissue is older. This underscores the need for biologically-aware ROI selection that considers both tissue age and implant behaviour. Notably, when the degradation behaviour of magnesium is controlled through alloying, various compositional and structural parameters of the surrounding bone can closely resemble those around titanium [23], suggesting that bone tissue outcomes are sensitive to the rate of material resorption. To enable meaningful comparisons across biomaterials, ROI selection must go beyond geometric proximity and instead reflect both the biological maturity of the tissue and the evolving behaviour of the material. Applying the same sampling strategies to fundamentally different materials risks misleading interpretation(s), as the tissue sampled may not be equivalent in age, composition, or origin. Without such context-aware approaches, assessments of bone quality and osteogenic performance are inherently flawed.

## 4 Physical, chemical, and biological properties of bone-repair biomaterials

**Fig. 1:**
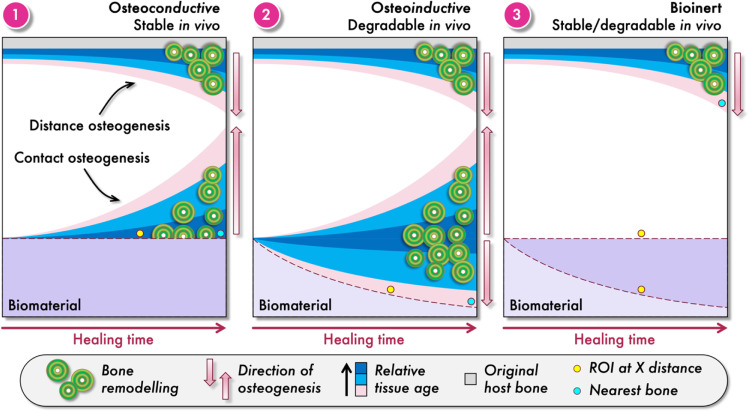
Schematic illustration of bone formation and remodelling as a function of healing time around three specific types of bone-repair biomaterials: (1) osteoconductive and stable, (2) osteoinductive and degradable, and (3) bioinert materials that may or may not maintain their external form *in vivo*. In all cases, distance osteogenesis originates from the osteotomy margin. Contact osteogenesis occurs only for osteoconductive and osteoinductive materials, but with a key distinction: for stable, osteoconductive materials, bone formation begins at the implant surface, such that the “nearest bone” corresponds to the oldest tissue. In contrast, the surface of degradable, osteoinductive materials evolves over time, therefore, the “nearest bone” is newly formed and represents the youngest tissue. Not all degradable materials are osteoinductive, and *vice versa*. Osteoinduction is typically confirmed by implantation at non-osseous sites, where distance osteogenesis does not occur. Bioinert materials do not support contact osteogenesis, hence, the implant surface remains separated from bone by unmineralised tissue. This difference in directionality and tissue maturation underscores the challenge of comparing materials based on region(s)-of-interest (ROIs) at fixed distances from the implant surface.

Mineralised, partially mineralised, and unmineralised areas comprise the bone-implant interface, which is a wide zone where physical and chemical interactions take place between the implant surface and the surrounding physiological system [24]. Various classes of biomaterials are used to repair bone defects unlikely to heal spontaneously. The properties of these biomaterials play a pivotal role in bone regeneration–some promote bone formation, while others have little or no intrinsic osteogenic activity. Materials used in bone repair can thus be broadly classified as osteo*conductive*, osteo*inductive*, or bioinert (Fig. 1). Osteoconductive materials provide a supportive substrate for existing bone to grow around and/or into the implant and make direct contact with the implant surface, whereas osteoinductive materials go further by recruiting osteoprogenitor cells, *i.e.*, mesenchymal stem cells, and promoting their differentiation into bone-forming cells, *i.e.*, the osteoblasts [25].

These distinctions have important implications for interpreting experimental outcomes. For example, in a heterotopic (non-osseous) implantation model, a titanium mesh alone shows minimal bone-implant contact, whereas the same mesh combined with a calcium phosphate yields abundant bone formation and markedly higher bone-implant contact [26]. While this may suggest superior material performance, the finding reflects the osteoinductive capability of the calcium phosphate in a setting where titanium, though osteoconductive in bone, cannot initiate bone formation. In a typical osseous site, where titanium supports contact osteogenesis, such disparity might not be observed. This highlights how the biological context, including implant site, can confound comparisons and influence the perceived outcomes.

In contrast, bioinert materials such as poly(ether ketone) (PEEK), polylactic acid (PLA), polycaprolactone (PCL), polytetrafluoroethylene (PTFE), and gold do not actively stimulate bone formation. Such materials typically do not support contact osteogenesis – the driving force behind *bone bonding* to artificial materials [27], but do not restrict bone ingrowth via distance osteogenesis [24]. PLA, for instance, has been shown to exhibit osteoconductive properties when functionalised with certain growth factors such as bone morphogenetic protein 2 (BMP-2) [28].

After implantation, bone-repair biomaterials are designed to either maintain their physical form and chemical characteristics or to degrade over time through bulk degradation and surface erosion [29]*.* Titanium (and titanium alloys), cobalt-chromium, PEEK, and PTFE are generally stable and do not undergo significant *in vivo* transformation. In contrast, PLA and PCL degrade slowly *in vivo*, and their degradation profiles can be tailored to suit specific clinical purposes, for example, faster resorption in non-load bearing sites or slower degradation where prolonged structural support is needed. Calcium phosphates such as hydroxy(l)apatite, monetite, and beta-tricalcium phosphate exhibit both physical degradation and chemical transformation towards more thermodynamically stable phases such as carbonated apatite. A similar process occurs with bioactive glasses. Magnesium-based implants [23], depending on their composition and degradation rate, may form tissue-contacting surface layers of magnesium oxides, hydroxides, or phosphates, and eventually calcium phosphate. The evolving surface chemistry and structural changes make it more difficult to generalise or predict bone formation patterns around degradable implants.

## 5 Two opposing directions of bone formation – contact and distance osteogenesis

Often comparisons are made across different types of biomaterials, and it is claimed that one is superior, despite dissimilar biological responses. An interpretation problem arises from the difference in the directional sequence of bone formation around different biomaterials [30], which influences where the youngest and the oldest bone is located relative to the implant. In stable (or permanent) materials such as titanium, titanium alloys, and cobalt-chromium, bone formation begins at the implant surface through contact osteogenesis, with lamellar bone extending outward over time. Degradable materials such as calcium phosphates also support contact osteogenesis, but as the implant degrades, new bone begins to occupy the resulting voids. In these cases, bone closest to the implant surface is typically younger and less mature, exhibiting lower mineral content, mineral crystallinity, and collagen cross-linking. Both material types also support distance osteogenesis, where bone grows inward from the osteotomy margin. Bioinert materials such as PEEK, PLA, and PCL do not support contact osteogenesis at all. Instead, bone forms only via distance osteogenesis, and the implant surface remains separated from the nearest bone by unmineralised tissue. These differing modes and directions of bone formation–contact osteogenesis *vs.* distance osteogenesis–mean that sampling the same radial distance from different biomaterials can yield bone tissues of vastly different age, structure, and biological maturity. To avoid misleading comparisons, it is critical to account for the biological directionality of bone formation when defining ROIs. Comparative studies should consider not only the distance from the implant, but also the origin and progression of bone ingrowth relative to material behaviour.

An *in vivo* model has recently been described that enables the simultaneous assessment of contact and distance osteogenesis around the same implant [31]. By allowing both modes to be observed within a single experimental setup, this approach helps to more accurately link bone formation patterns to material properties. Such models may also support more biologically relevant ROI selection, reducing the risk of misinterpretation when comparing materials with differing bioactivity or degradation behaviour.

Another often misunderstood aspect of bone-implant interactions is *stress shielding*, which refers to the reduction in physiological mechanical loading experienced by bone due to the presence of a stiff implant that bears most of the load [32]. This reduced mechanical stimulus can lead to bone resorption or limited bone formation around the implant, owing to the mechanoresponsiveness of osteocytes. Regions subjected to stress shielding may also experience reduced mechanostimulation, impairing recruitment of osteoprogenitor cells or maturation of newly formed bone. While not explicitly described in terms of contact or distance osteogenesis, stress shielding may influence both modes by modulating local mechanobiological signals critical for bone maintenance.

## 6 Conclusions

Bone-repair biomaterials–metals, ceramics, and polymers–exhibit diverse *in vivo* behaviours. Yet their evaluation is often hindered by inconsistent or biologically uninformed ROI selection, particularly when materials differ in degradation, bioactivity, or bone formation mechanisms. Fixed-distance ROIs may be appropriate for stable implants such as titanium, but this approach is unsuitable for degradable materials, where the implant boundary evolves over time. Likewise, comparing tissue formed through contact osteogenesis *vs.* distance osteogenesis may blur the distinction between biologically distinct processes, leading to flawed interpretations. Choosing the site of analytical interest at fixed distance from the implant surface does not guarantee reproducibility or biological relevance. particularly when materials vary in osteoconductivity, osteoinductivity, and degradation behaviour. The resulting tissue may differ not only in age and maturity but may also form through fundamentally different biological processes. Defining ROIs that follow the external contour of the implanted material may offer a more systematic and reproducible approach in the case of non-degradable materials [33]. However, for degradable materials, the changing geometry and bone ingrowth into the space initially occupied by the implant undermine spatial consistency across time points or between samples [34]. As biomaterials grow more complex, so do the challenges. A shift toward biologically-informed, standardised practices is urgently needed. Future studies must incorporate material-specific considerations into ROI selection, analytical design, or even the choice of *control* materials–each aligned with the dynamics of bone formation particular to the biomaterial being tested.

## CRediT authorship contribution statement

**Anders Palmquist:** Writing – original draft, Funding acquisition, Conceptualization. **Furqan A. Shah:** Writing – original draft, Funding acquisition, Conceptualization.

## Declaration of competing interest

The authors declare that they have no known competing financial interests or personal relationships that could have appeared to influence the work reported in this paper.

## Data Availability

No data was used for the research described in the article.
